# Challenges and Strategies for Developing Recombinant Vaccines against Leptospirosis: Role of Expression Platforms and Adjuvants in Achieving Protective Efficacy

**DOI:** 10.3390/pathogens12060787

**Published:** 2023-05-31

**Authors:** Natasha Rodrigues de Oliveira, Francisco Denis Souza Santos, Vitória Adrielly Catschor dos Santos, Mara Andrade Colares Maia, Thaís Larré Oliveira, Odir Antônio Dellagostin

**Affiliations:** Núcleo de Biotecnologia, Centro de Desenvolvimento Tecnológico, Universidade Federal de Pelotas, Pelotas 96010-610, RS, Brazil

**Keywords:** vaccine development, *Leptospira*, recombinant DNA technology, adjuvants, hamster model

## Abstract

The first leptospiral recombinant vaccine was developed in the late 1990s. Since then, progress in the fields of reverse vaccinology (RV) and structural vaccinology (SV) has significantly improved the identification of novel surface-exposed and conserved vaccine targets. However, developing recombinant vaccines for leptospirosis faces various challenges, including selecting the ideal expression platform or delivery system, assessing immunogenicity, selecting adjuvants, establishing vaccine formulation, demonstrating protective efficacy against lethal disease in homologous challenge, achieving full renal clearance using experimental models, and reproducibility of protective efficacy against heterologous challenge. In this review, we highlight the role of the expression/delivery system employed in studies based on the well-known LipL32 and leptospiral immunoglobulin-like (Lig) proteins, as well as the choice of adjuvants, as key factors to achieving the best vaccine performance in terms of protective efficacy against lethal infection and induction of sterile immunity.

## 1. Introduction

Leptospirosis is a neglected zoonotic disease with a worldwide distribution caused by pathogenic *Leptospira* spp. Infection occurs through direct contact with the environment (water and soil) that is contaminated with urine from bacteria-carrying animals [1]. It is estimated that more than one million human cases occur each year, affecting low-income populations in underdeveloped countries, especially where humans and animals live in close contact [2]. Clinical manifestations range from mild and nonspecific symptoms, such as fever and headache, to severe cases, such as Weil’s syndrome or severe pulmonary hemorrhage syndrome (SPHS), with mortality rates exceeding 70% [3]. In animals, leptospirosis is mainly associated with production losses and reproductive failure, leading to significant economic losses in the livestock sector [4,5].

Currently, the genus *Leptospira* has 69 species, of which at least 20 are pathogenic and have more than 260 serovars classified into 24 serogroups according to the serological classification based on the lipopolysaccharide (LPS) composition of the bacterial outer membrane [6,7]. Commercial vaccines against leptospirosis are available, especially for veterinary use, and consist of inactivated leptospires [4,8]. However, these formulations have well-known limitations, such as serovar-restriction protection and the induction of short-term immunity [9,10,11,12].

Studies have mainly focused on developing recombinant vaccines to overcome these issues [13]. Significant protective efficacy has been reported using different formulations; however, few studies have demonstrated the reproducibility of the efficacy results, the capacity to confer cross-protection in heterologous challenges, and the ability to induce sterilizing immunity [14,15]. Thus, the identification of novel surface-exposed and conserved proteins has been a promising strategy, especially propelled by advances in reverse and structural vaccinology, to select potential antigens that can confer cross-protection against different pathogenic serovars of *Leptospira* spp. [13,16]. Nevertheless, in most of these approaches, the vaccine antigen remains the only key to developing an effective vaccine against the disease.

**Figure 1:**
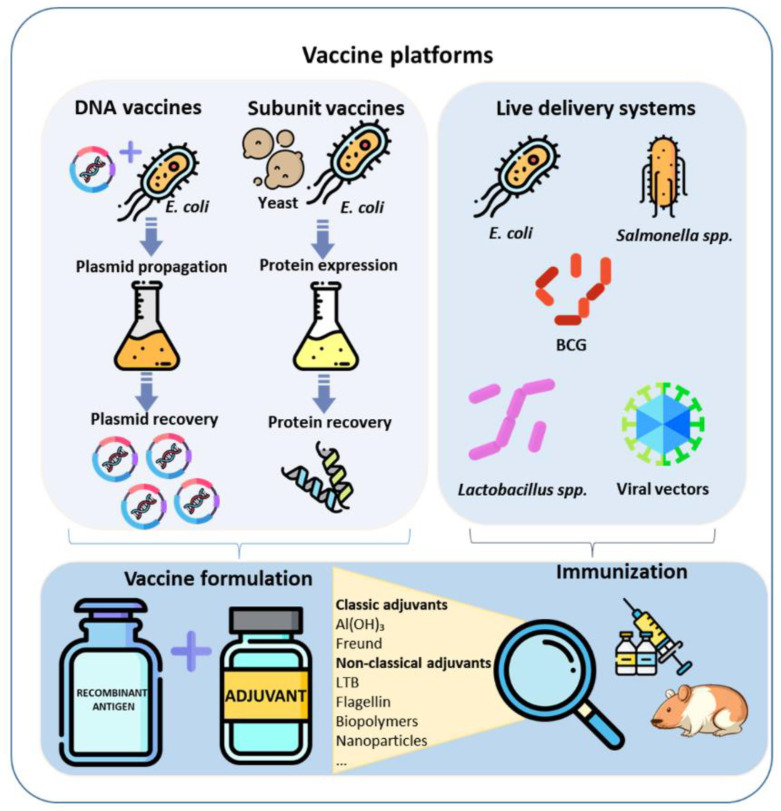
Vaccine platforms and adjuvants for the development of recombinant vaccines against leptospirosis. The most common antigen expression and delivery systems are shown. Classical and nonclassical adjuvants used to prepare vaccine formulations are highlighted. Al(OH)_3_: aluminum hydroxide; Freund: Freund’s adjuvant; LTB: heat-labile enterotoxin B subunit.

However, despite using the same vaccine antigens and similar administration schemes, different outcomes have been reported using distinct delivery platforms [14,15], highlighting the role of immune modulation in antigen delivery as another key factor in vaccine efficacy. A well-characterized protective immunological response would help in determining the best expression/delivery systems for the development of recombinant vaccines against leptospirosis. However, while there are correlates of protection associated with humoral immunity established for conventional vaccines (bacterins) against leptospirosis, none are available for recombinant vaccines [13,14,15]. Still, studies with hamsters, the main biomodel used in leptospirosis studies, are limited to humoral immunity analysis because reagents are not available to determine cellular immunity in this animal [13,14]. Considering these limitations, several strategies have been employed to produce and present leptospiral antigens in recombinant vaccine formulations against leptospirosis, including bacterial live vectors, microbial pattern receptor agonist molecules, and classic adjuvants [15]. In this review, our goal was to compare different expression platforms and delivery systems used in leptospirosis vaccine formulations. For this, a comprehensive literature search was performed using PubMed, Web of Science and Scopus databases. Titles, abstracts, or full text only from original articles were screened to select those that evaluated the protective efficacy of recombinant leptospirosis vaccines using specific antigens. The combination of the keywords “Leptospir*”, “vaccine”, and “recombinant”, was included for searching relevant studies using “AND” as a Boolean connector. The search was restricted to papers published in English. Finally, the articles were manually screened to seek out those that evaluated Lig and LipL32 antigens, followed by challenge experiments. Therefore, we presented here an overview of different expression and delivery systems employed to produce recombinant vaccines (Figure 1) based on the target antigens better described and explored in the leptospirosis research field, namely LipL32 and Lig proteins.

## 2. Bacteria-Based Expression Platforms

Bacterial expression systems are commonly used to obtain recombinant antigens due to their ease of handling, high level of expression capacity, fast growth rate, high productivity, short-run production, and reduced manufacturing cost [17,18]. *Escherichia coli*, a gram-negative, facultative anaerobic, rod-shaped, coliform bacterium, is widely used as a study model and is easy to engineer and adapt to new environmental conditions [19,20]. Despite extensive investigation of novel systems, *E. coli* remains the dominant host in use [21]. Reagents, equipment, and upstream processing for this platform have significantly lower costs than other expression systems [22]. However, there are some drawbacks to this system, including the absence of more complex post-translational modifications (PTMs), which can lead to the expression of misfolded, insoluble, or nonfunctional proteins [23]. Additionally, the manufacturing process should consider the removal of contaminant endotoxins, usually lipopolysaccharides, generated in this system. Although vaccines based on recombinant antigens offer several advantages over other vaccines, most of these present weak or poor immunogenicity when administered alone, thereby requiring the use of adjuvants to elicit a protective and long-lasting immune response [24].

Despite being the most abundant and highly conserved outer membrane protein in the entire leptospiral proteome, the lipoprotein LipL32 [25] cannot stimulate a protective immune response when purified from the *E. coli* expression system and used as a subunit vaccine [26,27]. However, coadministration with the highly immunogenic B subunit of *E. coli* heat-labile enterotoxin may enhance the immune-stimulating capability of LipL32, with protection ranging from 40 to 100% following a lethal challenge in hamsters [28]. Additionally, the LipL32 protein from serovar Autumnalis produced in *E. coli* and administered as a subunit vaccine in association with Freund or aluminum hydroxide with QS21 [29] provided 0–50% protection in gerbils challenged with *L. interrogans* serovar Canicola. This high variability in the range of vaccine protection obtained using different recombinant formulations makes LipL32 an enigmatic and controversial antigen.

Surface-exposed leptospiral immunoglobulin-like (Lig) proteins (LigA and LigB) are widely studied as vaccine candidates [30]. While LigB is highly conserved in pathogenic *Leptospira* sp., LigA is present only in *L. interrogans* and *L. kirschneri* strains. Vaccines using recombinant LigA and LigB have shown immunogenic potential, conferring a wide range of protection, and are able to protect animal models against lethal challenges with pathogenic *L. interrogans* [31,32]. Felix et al. (2020) [14] reviewed studies reporting the use of recombinant proteins based on the unique C-terminal region of LigA (LigANI) and the N-terminal portion of LigB (LigBrep), which is almost identical to that of LigA, and which has been used in different approaches to confer protection against leptospirosis. Da Cunha et al. (2019) [33] evaluated the immunoprotective activity of a chimeric fusion of LigA nonidentical fragment (LigAni) and LigB repetitive fragment (LigBrep), generating a construct named Lig chimera (LC). LC elicited a high humoral response with a 100% survival rate in a lethal challenge with *L. interrogans* strain Fiocruz L1-130. However, the authors also concluded that, despite the high levels of antibodies that vaccinated animals produced, sterilizing immunity was not achieved.

A recent study [34] has provided interesting results for new experiments by exploring the secreted exotoxins, so-called virulence modifying (VM) proteins, of the hypothetical PF07598 gene family. Mice immunized with recombinant *E. coli*-produced, endotoxin-free, leptospiral VM proteins demonstrate protective immunity against *L. interrogans* challenge, preventing the pathogenesis of clinical leptospirosis and leading to a marked reduction in leptospirosis target organ infections [34].

## 3. Yeast-Based Expression Platforms

The occurrence of PTMs has already been demonstrated for the LipL32 protein, which may be a barrier to generating effective vaccines from in vitro cultured bacteria because the candidate proteins are devoid of these modifications and the induced antibodies may not recognize the corresponding modified proteins expressed in the host [35]. Yeasts are attractive hosts for the expression of recombinant proteins owing to the advantages they have in common with prokaryotic systems, including fast growth, simple handling, high protein yield, low production cost, and knowledge of industrial production [36,37]. Furthermore, yeast systems may allow recombinant proteins to be correctly folded owing to their ability to perform PTMs of proteins in a manner similar to that used by eukaryotic cells, which is an advantage of eukaryotic systems [18]. In terms of safety, yeast systems can overcome limitations, as residual endotoxin accumulation associated with bacterial expression systems and viral contamination of mammalian expression systems are avoided [38]. However, yeasts also face some challenges because their glycosylation pattern is different from that of other micro-organisms, leading to excess mannose residues (hyperglycosylation) in recombinant proteins [39].

*Komagataella pastoris* has proven to be an excellent expression system for the production of recombinant LipL32 and LigAni from *L. interrogans*, showing excellent results with respect to the large-scale expression [40,41] without the need for subsequent solubilization and/or refolding steps. Vaccine formulations against leptospirosis using proteins expressed in *K. pastoris* have already been tested. Hartwig et al., 2014 [42], demonstrated that the LigAni protein secreted by *K. pastoris* only in the mannosylated form (mLigAni) protects hamsters as a subunit vaccine from lethal *L. interrogans* infection. However, sterilizing immunity was not achieved. Therefore, the use of yeasts offers advantages in terms of the secretion of target antigen for culture media, facilitating downstream steps, higher yield, and the possibility of post-translational modifications, such as glycosylation, that can enhance antigen immunogenicity [36,37]. However, the choice of expression system is mostly based on the group’s expertise and resources for downstream steps, particularly protein purification.

## 4. Live Recombinant Antigen Delivery Vehicles

Progress in molecular biology and genetic engineering fields has enabled the development of live recombinant vectors capable of delivering heterologous antigens and eliciting an immune response against their own antigens and the heterologous antigens [43,44]. Due to their ability to simulate natural infections [45], live recombinant vectors can stimulate humoral and/or cellular immune responses and can elicit mucosal immunity through oral administration [46,47]. However, despite the many advantages of using live bacteria as an alternative system for the delivery of heterologous antigens, safety concerns must also be considered [46].

### 4.1. Bacille Calmette-Guérin (BCG)

BCG was proposed as a recombinant vaccine vehicle for expressing heterologous antigens a long time ago because of its notable features. It is safe and has been administere billions of individuals with nonspecific immunostimulatory effects. BCG can be administered soon after birth, is highly immunogenic, and has prolonged persistence inside macrophages, thereby inducing long-lasting humoral and cellular immune responses. Moreover, it provides the possibility of generating T cell-mediated immunity against the cloned heterologous antigen [47,48].

Several studies have reported the use of recombinant BCG (rBCG) expressing foreign antigens from diverse pathogens. Seixas et al. (2007) [49] produced and characterized a rBCG-expressing LipL32 as an antigen against leptospirosis. Animals immunized with different constructs of rBCG/LipL32 showed the seroconversion of total anti-LipL32, with a higher titer than wild-type BCG, which was used as a control. Oliveira et al. (2019) [50] used rBCG in combination with a multiepitope protein approach based on the leptospiral antigens LipL32, LemA, and LigA (domains 11–13) to find a way to elicit a protective immune response and prevent renal colonization. Protective immunity induced by chimeric rBCG conferred 80–100% survival; no bacteria were detected in renal cultures, and qPCR data from the cultures were negative. Dorneles et al. (2020) [51] investigated the same antigens used in different chimeric constructs to transform BCG. Recently, Bettin et al. (2022) [52] developed an rBCG vectored vaccine expressing a chimeric antigen based on the TonB-dependent receptor (TBDR) epitopes (LIC10896, LIC10964, and LIC12374) from *L. interrogans*. Hamsters vaccinated with the rBCG:TBDRchi construct were fully protected from lethal leptospirosis, whereas the same recombinant protein as a subunit vaccine failed to protect animals (44.4% survival, *p* > 0.05; data not published). In studies performed by Oliveira et al. (2019) [50], Dorneles et al. (2020) [51], and Bettin et al. (2022) [52], it was shown that rBCG constructs were able to induce immune protection and prevent renal colonization against challenge with virulent *L. interrogans*. The combination of rBCG and chimeric multiepitope proteins appears to be a promising alternative against leptospirosis [53].

### 4.2. Lactobacillus

*Lactobacillus* species represent an attractive tool for vaccine production due to their generally regarded as safe (GRAS) status, reported adjuvant properties due to the peptidoglycan layer of some strains [54], and mucoadhesive ability, which is excellent for safe mucosal delivery vehicles of prophylactic and therapeutic molecules [55,56]. In addition, it is characterized by easy genetic manipulation and the availability of well-defined industrial production processes [57]. Different *Lactobacillus*-based vaccine prototypes have been developed and administered via the mucosal route, leading to both mucosal and systemic immune responses against expressed antigens [58]. A recent study showed that repeated oral administration of *L. plantarum*, a commensal probiotic and agonist of TLR-2 and NOD2, to C3H/HeJ mice mitigated acute leptospirosis and reduced renal lesions, although it did not prevent renal colonization against intraperitoneal infection with *L. interrogans* strain Fiocruz L1-130 [59].

Infected mice pretreated with *L. plantarum* [59] exhibited a 50% reduction in fibrosis and produced fewer transcripts of ColA1 than the negative control group (infected but pretreated with PBS), suggesting that oral treatment may have reduced the accumulation of collagen in the tubulointerstitial spaces, thereby preventing severe kidney pathology. Additionally, pretreatment with *L. plantarum* also induced the occurrence of mononuclear lymphocyte infiltrates, tubular damage, and higher interstitial nephritis scores than infected controls pretreated with PBS. The authors suggested that the pretreatment with *L. plantarum* in mice infected with *L. interrogans* triggers a complex myeloid and T-cell response that manages the deployment of monocytes from lymphoid tissue and the recruitment of neutrophils and macrophages to the kidney. Furthermore, the presence of myeloid cells in the kidney may be associated with a reduction in the observed pathogenesis. The use of *L. plantarum* as an immune modulator associated with a vaccine strategy against leptospirosis seems to be valuable and deserves further investigation [59].

### 4.3. Escherichia coli

*E. coli* can also be used as a delivery system, usually through the oral route. Oral approaches to delivering vaccines offer several advantages over other delivery systems, including convenience, cost-effectiveness, and the ability to induce both local and systemic immune responses, which have been well-described before [60]. Recently, an oral immunization based on a lipidated form of LigA using *E. coli* as a delivery system revealed a correlation between IgG levels and the survival of immunized hamsters [61]. The vaccine formulation was based on *E. coli* expressing a fusion of the OspA lipoprotein signal peptide with LigA immunoglobulin-like domains 7–13. The OspA signal peptide resulted in the lipidation of LigA and the incorporation of LigA 7–13 into the *E. coli* membrane fraction. This lipidation was able to modulate the immune response induced by oral immunization, as observed with the OspA formulation [62], which appeared to be important for overcoming oral tolerance by inducing a Th1/Th2 immune response [62].

Hamsters that were immunized by oral gavage with *E. coli* expressing the lipidated LigA7-13 antigen and challenged by intraperitoneal and intradermal routes developed a protective immune response to lethal challenges by *L. interrogans* serovar Copenhageni (37.5% and 62.5%, respectively). However, prevention against renal colonization was not observed [61]. In both experiments, LigA7-13-immunized animals that survived had higher antibody levels after 2 weeks of immunization than control-immunized animals. The natural adjuvant capabilities of *E. coli* and the lipidation of LigA7-13 may contribute independently to the production of highly immunogenic oral vaccines [61].

### 4.4. Salmonella

*Salmonella* is an intracellular pathogen that remains restricted to the endosomal compartment of eukaryotic cells and resists nonspecific killing mechanisms [63]. Recombinant antigens expressed in attenuated *Salmonella* strains can be delivered orally to mucosal surfaces, inducing a protective immune response against various targeted pathogens [64]. The invasive characteristics of *Salmonella* make it able to elicit B- and T-cell memory responses and confer upon *Salmonella* the significant potential to elicit long-lasting immunity. Samakchan et al. (2021) [65] evaluated the immune response of a recombinant attenuated *Salmonella* vaccine (RASV) prototype, NRSL32. This potential model was composed of an in-frame fusion between nucleotides encoding the N-terminal segment of the SspH2 effector protein containing the T3S signal and the leptospiral antigen LipL32. NRSL32 is an interesting candidate for the development of oral bacterial vectors [65].

The antigen delivery platform of RASV is based on the natural infection of intracellular *Salmonella*, which translocates virulence-effector proteins into host cells through T3SS. NRSL32 has demonstrated the ability to elicit effective immune responses by delivering LipL32 protein using SPI-2 T3SS [65]. In leptospirosis, the humoral immune response is the major protective immune mechanism against infection [66]. This RASV model stimulates adaptive humoral, cell-mediated, and mucosal immune responses. Significant titers of total IgG and IgA against rLipL32 were detected for a long time after vaccination. The stimulated antibodies were capable of specifically binding to LipL32 on the surface of pathogenic *Leptospira* spp. [65]. Moreover, this platform was capable of stimulating both Th1- and Th2-biased responses, although lethal challenge studies have not yet been conducted.

### 4.5. Viral Vectors

Viral vectors are based on modified viruses that can deliver genetic code for antigens to the target host. Several viral vectors are available for recombinant vaccine development, with differences in virion type, particle size, transgene capacity, and replicative cycle [67,68]. Viral vectors are characterized by their ability to induce cellular and potent antibody responses, high immunogenicity with intrinsic adjuvant properties, and the possibility of administering a single-dose schedule with long-lasting immunity [67,69]. Nevertheless, concerns about reduced effectiveness caused by previous exposure to the vector and the complexity of design and manufacture are challenges that need to be overcome [68]. Despite the many advantages associated with this system and its broad use for delivering antigens from specific pathogens for more than four decades [69], there is only one record of a study using this platform for leptospirosis vaccine development.

Branger et al. (2001) [70] produced a vectorized vaccine using recombinant adenovirus expressing the LipL32 protein from the serovar autumnalis, which provided 87% protection in gerbils challenged with *L. interrogans* serovar Canicola. In the same study, a similar OmpL1 adenovirus construct failed to protect animals. Despite the protection observed for the LipL32 adenovirus formulation, the negative control groups showed high survival rates (47–50%), which may indicate a sublethal challenge. It is likely that the limited use of viral platforms in leptospiral antigens relies on the difficulty of designing vector constructs, the high level of biosafety required, and other manufacturing challenges.

## 5. DNA Vaccines

Genetic vaccines utilize the genetic material of the microorganism that encodes the protein of interest [71,72]. To develop such vaccines, the target molecules are cloned into a plasmid that contains a strong eukaryotic cell promoter, a poly(A) signal sequence, and a selectable marker [18,71,72,73]. Then, the recombinant molecule is associated with an adjuvant/costimulator and inserted directly into the host cells. RNA vaccines are a relatively recent technology, and there are no published articles about their use in the development of leptospirosis vaccines. Instead, DNA vaccines have been widely explored for this purpose [73].

Recombinant DNA vaccines that use plasmid vectors encoding LipL32 from serovar autumnalis or LipL32 from serovar Grippotyphosa provide 60% protection in animals challenged with the same virulent strain [29]. DNA vaccines carrying the LigBrep portion (LigA identical domains 1–6) using aluminum hydroxide as an adjuvant protected 40–62.5% of hamsters challenged, whereas the LigBni portion (nonidentical domains 7–12) was not protective using the same presentation as the DNA vaccine. The DNA prime-boost strategy for the LigBrep portion improved protective efficacy levels to 83.3% [32]. However, unlike LigBrep, no protective effect was achieved with DNA vaccines based on the LigAni portion alone [74]. The chimera comprising the LigAni and LigBrep fragments, constructed by da Cunha et al. (2019) [33], was able to protect 100% of the hamsters vaccinated with a prime-boost strategy, whereas only partial protection (25%) was obtained with the DNA vaccine alone. Significant results regarding the protective efficacy were also obtained using LemA antigen as a DNA vaccine (62.5%) or as part of a prime-boost strategy (87.5%) after homologous challenge in the hamster model [75]. The leptospiral recombinase A (RecA) and flagellar hook-associated protein (FliD) antigens evaluated as DNA vaccines (62.5%) or as part of a prime-boost strategy (~91–100% protection) in homologous or heterologous challenge also showed significant protective efficacy [76].

Considering most of the results with leptospiral vaccines [32,33,75,76], it is clear that the prime-boost strategy has more potential to induce a mixed Th1/Th2 response and, consequently, better protection results than DNA vaccines alone. To optimize DNA vaccine platforms to increase antigen immunogenicity, efforts have been made to improve DNA delivery techniques and the choice of adjuvants for coadministration [71]. Ongoing leptospirosis studies include the production of formulations with nonclassical adjuvants such as the use of polyinosinic-polycytidylic acid (poly I:C), a ligand for endosomal TLR3, for coadministration with LipL32 DNA vaccine [77]; the development of a unique DNA plasmid expressing both LipL32 and Loa22 antigens as DNA-encapsulated chitosan nanoparticles [78]; LipL32 DNA vaccines tagged with EGFP and hGMCSF adjuvant conjugated to chitosan, Bacopa saponin, or tripolyphosphate nanoparticles [79]; or the association of probiotics, such as *Saccharomyces boulardii* [73]. Despite these promising results, further studies should be conducted to investigate whether these vaccines confer protection against lethal challenges.

## 6. Commercial Adjuvants

### 6.1. Aluminum Hydroxide

Adjuvants are important components of vaccines that can stimulate the host immune response against antigens and induce an efficient protective response [80,81]. These substances can enhance the strength and longevity of immune responses and influence the type of response [82]. The use of adjuvants can reduce the amount of antigen required per vaccination, potentially reducing side effects at the inoculation site [83]. Aluminum salts such as aluminum hydroxide and aluminum phosphate were the first licensed adjuvants and are widely used in veterinary and human vaccines [84]. Currently, it is known that these adjuvants stimulate cells of the innate immune system by activating the NLRP3 inflammasome pathway, releasing signaling molecules called damage-associated molecular patterns (DAMPs), and inducing the differentiation of naive T-cells into Th2 cells, thereby developing a Th2-type immune response [85,86,87]. Some studies have shown that when aluminum salts were tested in a few experimental DNA vaccines against viral antigens, aluminum hydroxide presented an inhibiting effect, whereas aluminum phosphate adjuvant increased the humoral and cellular immune response against these antigens [88,89]. However, further studies are needed to better understand these effects with other antigens.

Aluminum hydroxide, the most common adjuvant in the formulation of recombinant vaccines against leptospirosis, has a successful record of use in animal experimentation where it promotes a significant antibody response, but with variable results in protection and renal sterility [14,15]. Several vaccine formulations containing recombinant adhesins, LigA and LigB proteins, have been evaluated due to their important roles in virulence and immunity [13,15]. Vaccines using different recombinant versions of LigA and LigB associated with aluminum hydroxide stimulate the humoral immune response and protect against pathogenic *Leptospira*. Some studies have shown that formulations with LigA elicit protective efficacy of 67% and 100%.

Vaccines formulated with three different recombinant LigB fragments and aluminum hydroxide (rVarB2, rVarB1, and rLigBcon) resulted in protective efficacy of 33%, 54%, and 71%, respectively. When these three fragments were administered together, the protective efficacy of the vaccine was enhanced to 83% [90]. Another study showed that a recombinant vaccine based on a conserved region of LigB associated with aluminum hydroxide stimulated high levels of protection (80.0–100%) and sterilizing immunity (87.5–100%) among vaccinated survivors [91]. A recombinant Lig chimera composed of LigA and LigB fragments (LigAni and LigBrep) was constructed, and a vaccine formulated with aluminum hydroxide conferred 100% protection against challenge; however, sterilizing immunity was not achieved [33]. Another study showed that the chimeric protein based on the amino acid sequences of five outer membrane proteins (OMPs) (LigA, Mce, Lsa45, mpL1, and LipL41) associated with aluminum hydroxide promotes only 50% protection against infection and does not promote full clearance of bacteria in the kidneys of animals [92].

Most of the recombinant vaccines reviewed here that utilized aluminum hydroxide showed variable protection against leptospirosis and did not promote sterilizing immunity. However, in cases where the recombinant vaccine conferred protection and more than one adjuvant was tested, the formulation containing aluminum hydroxide consistently induced a protective immune response [14]. Given that aluminum hydroxide is the most widely used adjuvant in human and veterinary vaccines, it should be considered for use in formulations with recombinant proteins to develop leptospirosis vaccines [14].

### 6.2. Freund’s Adjuvant

Freund’s adjuvant (FA) is a water-and-oil emulsion-based adjuvant that is widely used as a potent stimulator of the immune response in antibody production vaccination protocols [93,94]. The mechanism of action is based on oily antigen deposits, from which the antigen is continuously released at the injection site. This leads to an increase in the antigen’s lifetime and induces a strong local innate immune response mediated by phagocytosis, leukocyte recruitment and infiltration, and cytokine production [95]. FA has two different formulations, the complete Freund’s adjuvant (CFA), which consists of heat-killed *Mycobacterium tuberculosis* emulsified in paraffin oil, and the incomplete Freund’s adjuvant (IFA), which has the same formulation without *M. tuberculosis*. FA stimulates a strong immune response, but it is highly toxic and can cause tissue necrosis at injection sites, in particular CFA [96,97]. Thus, CFA is frequently used for initial immunization, and booster doses are administered with IFA to decrease these adverse effects [97].

FA has been successfully used in the formulation of recombinant leptospiral vaccines. The vaccine composed of the carboxy-terminal portion of LigA plus FA, CFA on the first dose, and a second boost of antigen in IFA induced robust IgG responses and conferred immunoprotection against lethal challenge (67–100%) in the hamster model, but this immunization protocol did not confer sterilizing immunity [98]. Coutinho et al. (2011) [99] demonstrated that a purified recombinant subunit vaccine composed of a LigA three-domain region and FA protected hamsters from lethal infection with 100% protection; however, sterilizing immunity was not observed. Another study evaluated a recombinant subunit vaccine formulated with domains of LigA, LigB, or a combination of LigA and LigB in FA. The results showed that LigA protected hamsters against lethal infection but not renal colonization, and immunization with LigB, either alone or in combination with LigA, did not confer sterilizing immunity [100].

FA is widely used in the formulation of leptospiral vaccines. However, CFA is reported to cause pain and distress in animals owing to its high reactogenicity [101]. Thus, there are many recommendations and regulatory issues related to the use of CFA, and alternative adjuvants are recommended [93].

## 7. Pathogen Agonists and Other Adjuvants

### 7.1. Pathogen Agonists

Pathogen-agonist adjuvants are bacteria-derived components that include molecules functioning as pathogen-associated molecular patterns (PAMPs) and DAMPs that are agonists of innate immune cell receptors, such as Toll-like receptors (TLR) [102,103]. These adjuvants are also known as TLR agonist-based adjuvants [104]. The activation of TLRs on DCs using highly conserved PAMPs in microbes stimulates antigen-specific T and B cell responses [105]. These molecules have been tested as adjuvants in recombinant leptospiral vaccines. The monophosphoryl lipid A (MPLA) from *Bordetella pertussis* was used in association with a chimeric protein based on the amino acid sequences of OMPs LigA, Mce, Lsa45, mpL1, and LipL4, and induced only 55% protection against infection and did not promote renal sterilizing immunity. Flagellin is a protein subunit of the flagellar filament, expressed mainly by *Salmonella* [106], and is an agonist of TLR5 [107]. The flagellin (FliC) from *S. enterica* serovar Typhimurium has been used in combination with the carboxy-terminal portion of LigA (LigAC) and a pool of six different OMPs (Lp11, Lp21, Lp22, Lp25, Lsa30, and Lp35) in a recombinant vaccine that conferred 93% protection for LigAC plus FliC and 86% for LigAC, Lp pool, and FliC against lethal challenge but did not promote sterilizing immunity [108].

Xanthan gum is a high-molecular-weight extracellular polysaccharide produced through fermentation of the phytopathogen *Xanthomonas* spp. [109]. Xanthan has a backbone chain consisting of (1,4) β-D-glucan cellulose and contains mannose in its structure; thus, it may be recognized by both TLR-4 and TLR-2, respectively, which stimulate the production of inflammatory cytokines, including TNF-α and IL-12 p40, via nuclear factor-κB (NFκB), which is sufficient to trigger the innate and adaptive immune responses [110,111,112]. A subunit vaccine composed of LigA and xanthan gum induced a strong antibody response and a high protective efficacy (100%) against lethal challenge [113]. Synthetic oligodeoxynucleotides (ODNs) expressing unmethylated CpG motifs mimic bacterial DNA and act as TLR-9 agonists that interact with innate immune cells. In addition, CpG ODN-mediated activation stimulates antibody production and T-cell responses to a variety of protein antigens. The combination of LigA with CpG and xanthan also conferred 100% protection against lethal challenges [113]. However, the combination of LigANI and CpG ODNs was unable to induce an IgG response and promoted a nonsignificant level of protection (17%) [114].

### 7.2. The B Subunit of the Heat-Labile Toxin of E. coli (LTB)

The B subunit of *E. coli* heat-labile enterotoxin (LTB) has immunomodulatory characteristics and potential as a mucosal and parenteral vaccine adjuvant [115]. LTB stimulates a robust immune response, possibly because it induces the expression of MHC class II and costimulatory molecules, such as B7, CD40, CD25, and ICAM-1, on B cells. LTB enhances B7-2 expression on antigen-presenting cells, mainly DCs, leading to the costimulation of CD4+ T cells [116]. In a hamster model of leptospirosis, the recombinant LipL32 coupled to (LTB-LipL32) or coadministered with LTB provided survival rates ranging from 40% to 100% against lethal challenge [28]. More recently, Ghazali-Bina et al. (2019) [117] reported promising results with the recombinant proteins LcpA and LenA in combination with LTB. Hamsters immunized with rLenA-plus-rLTB, rLcpA-plus-rLTB, and rLenA-plus-rLcpA-plus-rLTB proteins showed 60%, 74%, and 80% survival rates, respectively.

### 7.3. Carbon and Halloysite Clay Nanotubes

Nanotubes can serve as adjuvants and carriers in vaccines, delivering biomolecules such as proteins and peptides to antigen-presenting cells (APCs), particularly dendritic cells (DCs), which are essential for stimulating a potent immune response [118,119,120]. Moreover, nanotubes can act as antigen depots, prolonging immune activation [118]. Carbon nanotubes (CNTs) are entirely created from carbon atoms and can be single-walled, double-walled, or multiwalled (MWCNTs) [121]. Halloysite clay nanotubes (HNTs), on the other hand, are hollow tubular structures composed of clay material that can be mined from deposits.

In a study by Oliveira et al. (2016) [114], the use of carbon nanotubes as adjuvants in subunit vaccines against leptospirosis was tested. Immunization with LigANI and COOH-MWCNTs elicited a high level of IgG antibodies but was not protective. In another study, Hartwig et al. (2015) [121] evaluated the feasibility of using HNTs and carboxyl-functionalized MWCNTs (COOH-MWCNTs) as antigen carriers for recombinant LipL32 (rLipL32). Although both formulations stimulated an increase in IgG antibody levels, none of them conferred protection against lethal challenges.

## 8. Current Gaps and Future Directions

Despite the discovery and evaluation of new targets, classical surface proteins such as the lipoprotein LipL32 and Lig proteins (LigA and LigB) remain the most studied antigens in the field of recombinant vaccine development against leptospirosis [113,114] (Table 1). These proteins were among the first leptospiral antigens identified using classic biotechnology, and the broad availability of data produced in the last two decades (structural, functional, and immunogenicity data) justifies their frequent selection as vaccine targets. Analyzing studies that focus on these classical antigens, subunit vaccines using *E. coli* as a heterologous system is the preferred expression platform for most research groups. This preference is likely due to the broad range of protocols available, ease of handling, and low cost of production compared to other expression systems or delivery platforms. Additionally, the option of subunit vaccines allows for greater combinations with adjuvants compared to other types of vaccines, which may be preferable for initial clinical trials.

Although the selection of an ideal adjuvant for a vaccine trial should depend on the type of immunological modulation desired against the target pathogen, aluminum hydroxide has been the most commonly used adjuvant in vaccine efficacy tests with recombinant leptospiral antigens [14]. Interestingly, data from different research groups using the same antigens as subunit vaccines formulated with different adjuvants have demonstrated divergent protection results [83]. Subunit vaccines using the rLigAni antigen have shown protective efficacy ranging from 60–100% using aluminum hydroxide or other adjuvants like Freund, Xanthan, LMQ, Montanide, and AS04. A recent study [124] analyzed the protective efficacy and the immunological responses induced by subunit formulations using rLigA with aluminum hydroxide compared to AS04 and Montanide ISA720VG clinical adjuvants. As a result, formulations with both AS04 and Montanide ISA720VG adjuvants induced superior immune responses (67% and 83%, respectively) and protective efficacy compared to aluminum hydroxide (50%), but sterilizing immunity was still not achieved with any adjuvant. In the absence of an immune correlate, the selection of a single adjuvant, such as aluminum hydroxide, for initial screening with several antigens, as in reverse vaccinology studies, can be an interesting option for experimental design, reducing animal use and costs [14]. However, after the identification of antigens that present at least partial protective efficacy in initial trials, it is important to continue investing in formulation improvement, especially by testing different adjuvants, which can significantly improve vaccine efficacy.

Another classical antigen that has been highlighted by conflicting results as a subunit vaccine is LigB. The rLigBrep fraction administered with an aluminum hydroxide adjuvant conferred 80–100% protection in the hamster model with sterilizing immunity [91]. However, another study [100] showed that LigB administered with Freund’s adjuvant (complete and incomplete) failed to protect hamsters against a lethal challenge with *L. interrogans*. The lack of reproducibility of subunit vaccines based on rLigBrep among independent research groups is possibly due to differences in protocols applied, where maximum effectiveness was achieved with the hydroxide aluminum formulation by intramuscular administration. A comparative study of the rLigBrep antigen using different adjuvants, as was performed with LigAni [124], would help to elucidate the immunological basis of this antigen’s effectiveness.

Despite the great availability of data, the different immunization protocols (route of administration, number of doses, amount of antigen applied, adjuvant, challenge strain, etc.) in these studies make it difficult to conduct a rational analysis about the best formulation to choose. Although some studies present promising results, failures in experimental challenges using virulent *Leptospira* strains are recurrent, resulting in a high rate of survivors in the negative control group (usually administered with saline alone or combined with an adjuvant), which has reached up to 60% (Table 1). Another recurring bias is the lack of standardization of challenge doses applied (ranging from 2 to 500 × LD_50_ in studies described in Table 1) or the absence of information in the Methods section about equivalent LD_50_ doses applied. Additionally, many studies used too few animals per group (<10), which is associated with the application of subclinical challenge doses that could compromise the real vaccine efficacy. In view of the protocol variability, it is also important to consider the quality of the antigens and formulations produced for each study. Ptak et al. (2019) [125] demonstrated that the stability of several Lig protein Ig-like domains is affected by variations in physiological temperatures, highlighting the importance of considering thermostability parameters for vaccine production [125]. Taking subunit vaccines based on Lig proteins as an example, the variability of antigen regions selected for each study (Table 1), which can include poor thermostability, could be another important bias that justifies the several inconsistent results observed so far.

The incorporation of biophysical and structural methods into the vaccine design process, as well as the adoption of good practices in vaccine efficacy testing for recombinant formulations (such as those used in bacterin efficacy testing), can help reduce experimental errors. One example of good practice is the use of statistical analysis, such as the Fisher test, to determine the number of animals required per group. Another is the use of challenge doses within the requirements of the 9 CFR (10–10,000 LD_50_ equivalents), as well as the maintenance of stable virulent *Leptospira* strains for use in challenge experiments.

Based on literature reports of a lack of reproducibility in recombinant vaccine efficacy tests using hamsters, some authors [126,127] speculate on the hamster model’s suitability and propose the consideration of alternatives such as acute and chronic mouse models in the evaluation of vaccine candidates. However, mouse susceptibility to lethal infection is conditional on the use of high doses of the inoculum of *Leptospira* spp. (~10^6^–10^8^ compared to ~10^2^ and 10^3^ for the hamster model) and the availability of mutant strains, like the C3H/HeJ, which still limits its wide applicability.

A good vaccine for leptospirosis should be able to protect against lethal disease and induce sterile immunity against representative serovars of human and animal infections [13,14,16]. Despite the significant protection achieved in several experiments using LipL32 or Lig antigens delivered alone or in combination on different platforms, until now, only vectorized vaccines based on rBCG and a single rLigBrep subunit vaccine were able to confer full protection in terms of survival, tissue lesions, and renal clearance, whereas animals vaccinated with other vaccine approaches were protected only against death. It is known that BCG induces a robust Th1 response characterized by the massive presence of Th17 and cytotoxic T lymphocytes [48]. Although the mechanisms involved in the heterologous protective effects of BCG are not yet fully understood, recent studies have shown that BCG is able to trigger innate immune memory mechanisms, also known as “trained immunity”. These reprogramming cells may act as APC (antigen-presenting cells) and augment T-cell immunity by inducing IFN-γ production and subsequent Th1 cellular responses [128]. This protective immunological modulation characterized by the predominant induction of a cellular response involving IFN-γ induction has been observed with other vectorized vaccines based on rBCG applied to bacterial and viral diseases (reviewed by Mouhoub et al., 2021 [129]). Additionally, the kind of immunological response stimulated by BCG also corroborates with the protective efficacy elucidated by some leptospirosis monovalent bacterins used in cattle, which are marked by significant IFN-γ production [130,131].

Although beneficial effects of trained immunity on leptospirosis have been reported through host-directed treatment using a TLR2/NOD2 agonist (CL429) [132] and proposed as an adjuvant to increase the efficiency of leptospiral vaccines, deeper studies are needed to characterize the type of immune modulation induced by rBCG expressing leptospiral antigens. In the future, comparative analysis of BCG-based formulations and other delivery systems, as well as whole-cell inactivated vaccines, will allow for a better comprehension of the immune mechanisms that mediate the protective efficacy of BCG as a vaccine vector for leptospirosis. However, an important limitation in this field is the shortage of supplies and kits to analyze cellular immune responses in the hamster model [126]. This restriction limits the studies to antibody-level determination and gene expression analysis, making it difficult to deepen the understanding of the immune responses that mediate complete protection against this disease. Additionally, the lack of an immune correlate means that vaccine efficacy determination depends exclusively on the use of experimental animals, with the hamster as the main biomodel for this purpose. Thus, it is necessary to invest in the development of immunological supplies for the analysis of vaccine efficacy in hamsters as experimental animals and, consequently, to improve the comprehension of protective responses for leptospirosis.

However, the development of leptospiral recombinant vaccines is still limited by the lack of results regarding cross-protection against different serovars. The reason for the lack of publication of heterologous protection data is not clear, but we believe it may be attributed to the difficulty in maintaining virulent strains of *Leptospira* spp. in rodent models or to the greater interest of research groups in focusing on the improvement of protective formulations using the homologous challenge as a reference. The validation of the ability to provide heterologous protection by the most effective vaccine approaches obtained until now will allow significant improvements focused on the characterization of protective immunity and vaccine biosafety aspects. Based on the data presented here, we believe that the better approach would be to invest in trials of comparative analysis using different expression platforms (subunit, DNA, or live), focusing on the performance analysis of one or only a few antigens. This approach could decrease the risk of discarding a good vaccine target based only on a negative result obtained by the choice of a specific expression platform or delivery system. Notably, this choice should consider some rational aspects like the target species for which the vaccine is developed (e.g., the use of BCG as a vaccine vector would compromise the diagnosis of tuberculosis in bovines), safety issues (use of attenuated live vaccines for immunocompromised individuals), the antigen’s size and toxicity, as well as its levels and stability of expression in vitro and in vivo. In the field of recombinant live vaccines, studies using rBCG are predominant, but the use of *Salmonella* and yeasts has been proposed as promising. When testing this broad approach is not initially available, investment in subunit vaccines expressed by *E. coli* seems to be the most feasible and practical way to start efficacy tests. Furthermore, in view of interesting results with one or more platforms (especially on protection against death and renal colonization), efforts should be focused on formulation improvements, such as tests with different adjuvants, doses, or routes of administration, and heterologous challenges.

In conclusion, the search for a universal vaccine against leptospirosis has been based on protective antigens delivered on a platform able to induce cross-protection and efficient immunological memory. Although some studies have shown promising results with cytoplasmic antigens [76], secreted proteins [34], or nonconventional vaccine antigens [133], until now, OMPs remain the best option for antigen choice in order to achieve these goals; they are surface-exposed proteins and well conserved among pathogenic species of *Leptospira*. Recently, other OMPs have been evaluated as potential vaccine antigens against leptospirosis [124,126,127,134]. However, despite the development of several formulations, the reproducibility of their immunoprotective potential needs to be evaluated in additional experiments. Finally, the findings observed for the classical proteins reviewed here emphasize the importance of selecting the ideal expression/delivery system as the key factor in achieving optimal antigen performance, which directly impacts vaccine efficacy and the induction of sterilizing immunity, even for antigens with low performance in other vaccine presentations.

## Figures and Tables

**Table 1:** Vaccine approaches and immunoprotection data obtained for the most extensively studied leptospiral antigens.

Vaccine Approaches					Challenge Parameters			Protection Outcomes					
Antigen	Expression/ Delivery Platform	Dose ^a^	Adjuvant ^b^	Via ^c^	Model	LD_50_^d^	Type ^e^	Vaccine Protection ^f^	Negative Control Lethality ^g^	Sterile Immunity		Experiment Repetitions ^h^	Ref.
										Culture	qPCR		
LipL32													
LipL32_full_	Adenovirus (Vectorized)	3×/10^9^ PFU/21 d	NA	IM	Gerbils	NA	HE	86.6% (26/30)	51% (17/33)	NA	NA	2	[70]
	*E. coli* (Subunit)	3×/50 µg/14 d	FA	SC	Gerbils	NA	HE	0% (0/15)	86.6% (13/15)	NA	NA	2	[29]
			Alum + QS21					50% (7/14)	40% (6/15)				
	pcDNA (aut) (DNA)	2×/100 µg/21 d	NA	IM				60% (9/15)	65% (13/20)				
	pcDNA (grip) (DNA)		NA										
LipL32_full_	*E. coli* (Subunit)	2×/43.5 µg/14 d	LTB	IM	Hamster	5×	HO	80–87% (12/15–13/15)	60–73% (9/15–11/15)	NA	NA	3	[28]
LipL32_full_	rBCG (Vectorized)	2×/10^6^ CFU/21 d	NA	IP	Hamster	NA	HO	12.5–55.9% (1/8–19/34)	88% (30/34)	Yes	NA	3	[49]
LipL32_full_	*E. coli* (Subunit)	1×/868 pmol	Alum	SC	Hamster	NA	HO	20% (1/5)	100% (5/5)	Yes	NA	1	[27]
LipL32_full_	*E. coli* (Subunit)	2×/30 µg/14 d	Alum	NA	Hamster	NA	HO	0% (0/10)	80–100% (2/10–10/10)	NA	NA	1	[26]
LipL32_155–200_													
LipL32_full_	*E. coli* (Subunit)	2×/50 µg/14 d	Alum	NA	Hamster	NA	HO	0% (0/6)	NA	NA	NA	1	[121]
			COOH-MWCNTs										
			HNTs										
LipL32_full_	rBCG (Vectorized)	2×/10^6^ CFU/21 d	NA	SC	Hamster	5×	HO	90–100% (9/10–10/10)	100% (10/10)	Yes	Yes	2	[51]
Chimeras													
LemA_28–157_-LigAni_943–1224_ LipL32_224–272_-LemA_28–157_	rBCG (Vectorized)	2×/10^6^ CFU/21 d	NA	SC	Hamster	5×	HO	100% (10/10)	100% (10/10)	Yes	Yes	2	[51]
								100% (10/10)				2	
LipL32_224–272_-LemA_28–157_-LigAni_943–1224_	rBCG (Vectorized)	2×/10^6^ CFU/21 d	NA	SC	Hamster	5×	HO	80–100% (8/10–10/10)	100% (10/10)	Yes	Yes	1	[50]
LigAni_943–1224_-LigBrep_131–645_	*E. coli* (Subunit) *E. coli* (Subunit) pTARGET-chimera (DNA) Prime-boost *	2×/50 µg/14 d	Alum	IM	Hamster	5×	HO	100% (8/8)	100% (8/8)	No	No	1	[33]
		2×/50 µg/14 d	ISA 50 V2					100% (8/8)					
		2×/100 µg/14 d	NA					25% (2/8)					
		2×/100 µg DNA + 50 µg protein/14 d	Alum (boost)					100% (8/8)					
LigAni_631–1224_-LigBrep_19–672_	*E. coli* (Subunit)	3×/100 µg/14 d	FA	SC	Hamster	500×	HO	100% (8/8)	100% (5/5–6/6)	No	NA	3	[100]
LigAni_852–1107_- Mce_131–207_- Lsa45_190–250_- OmpL1_153–221_- LipL41_213–276_	*E. coli* (Subunit)	2×/50 µg/14 d	Alum	SC	Hamster	NA	HO	33–50% (3/9 –3/6)	80–100% (8/10–6/6)	No	NA	2	[92]
			MPLA					50–60% (3/6 –6/10)					
			DDA					0% (0/10)					
OmpL1_87–98; 173–191_-LipL32_133–160; 201–218_- LipL21_97–112; 176–184_	*E. coli* (Subunit)	3×/200 µg/14 d	Alum	SC	guinea pigs	2×	HO	80% (4/5)	100% (5/5)	Yes	NA	1	[122]
Ligs													
LigAni_625–1224_	*E. coli* (Subunit)	2×/50 µg/14 d	NA	SC	Hamster	5×	HO	0% (0/12)	100% (12/12)	NA	NA	2	[114]
			Alum					67% (8/12)					
			COOH-MWCNTs					0% (0/12)					
			CpG ODNs					17% (2/12)					
			COOH-MWCNTs + CpG ODNs					17% (2/12)					
LigAni_625–1229_	*E. coli* (Subunit)	2×/50 µg/14 d	NA Alum CpG Xanthan Xanthan + CpG	SC	Hamster	36×	HO	0% (0/6) 66.7% (4/6) 16.7% (1/6) 100% (6/6) 100% (6/6)	83.3–100% (5/6–6/6)	No	NA	2	[113]
LigAni_629–1229_	*E. coli* (Subunit)	2×/50 µg/15 d	Alum	SC	Hamster	100×	HO	100% (10/10)	70–100% (7/10–10/10)	No	NA	2	[108]
LigAni_629–1224_	*E. coli* (Subunit)	3×/20 µg/14 d	LMQ	SC/ IM	Hamster	20×	HO	60% (3/5)	100% (5/5)	No	NA	1	[123]
LigAni_631–1224_	*E. coli* (Subunit)	3×/100 µg/14 d	FA	SC	Hamster	500×	HO	100% (8/8)	100% (5/5–6/6)	No	No	3	[100]
LigAni_631–1224_	*E. coli* (Subunit)	3×/100 µg/14 d	FA	SC	Hamster	NA	HO	100% (8/8)	100% (0/8)	No	NA	2	[99]
LigAni_631–1033_								50% (4/8)					
LigAni_631–851_								0% (0/8)					
LigAni_852–1224_								100% (8/8)					
LigAni_852–1124_								100% (8/8)					
LigAni_943–1224_								100% (8/8)					
LigAni_943–1124_								25% (2/8)					
LigAni_1034–1224_								50% (4/8)					
LigAni_624–1224_	rBCG (Vectorized)	2×/10^6^ CFU/21 d	NA	SC	Hamster	5×	HO	100% (10/10)	100% (10/10)	Yes	Yes	2	[51]
LigA-LAV (domains 8-13)	*E. coli* (Subunit)	2×/50–25 µg/21 d	Alum AS04 Montanide	SC	Hamster	100×	HO	50% (3/6) 67% (4/6) 83% (5/6)	100% (6/6)	NA	No	3	[124]
LigAni_629–1229_ LigBni_629–1112_ LigBrep_1–628_	pTARGET-chimera (DNA)	2×/100 µg/21 d	Alum	IM	Hamster	5×	HE	0% (0/8)	100% (6/6)	No No Yes	NA	1	[74]
								0% (0/8)					
								62.5% (5/8)					
LigBrep_19–672_	*E. coli* (Subunit)	3×/100 µg/14 d	FA	SC	Hamster	500×	HO	37.5% (3/8)	100% (6/6–8/8)	No	No	3	[100]
LigBrep_131–645_	*E. coli* (Subunit)	2×/20–100 µg/14 d	Alum	IM	Hamster	10×	HO	85.7–100% (8/10–10/10)	70–100% (7/10–10/10)	Yes	Yes	7	[91]
LigBrep_1–628_	*E. coli* (Subunit) pTARGET (DNA) pTARGET (DNA) Prime-boost *	2×/100 µg/21 d	Alum	IM	Hamster	5×	HE	0% (0/6)	100% (5/5)	Yes	NA	1	[32]
			Alum					40% (2/5)					
			NA					0% (0/6)					
			Alum					83.3% (5/6)					

^a^ Dose: number of doses applied, the antigen dose in the vaccine formulation and interval between doses are shown in sequence and separated by slashes. PFU: plaque-forming unit; CFU: colony-forming unit. ^b^ Adjuvant type: Alum: aluminum hydroxide; FA: Freund’s adjuvant: first dose applied with complete Freund’s adjuvant and subsequent doses with incomplete Freund’s adjuvant; QS21: purified *Quillaja saponaria* vaccine adjuvant; HNTs: halloysite clay nanotubes; COOH-MWCNTs: carboxyl-functionalized multiwalled carbon nanotubes; CpG ODN: CpG oligodeoxynucleotides; ISA 50 V2: Montanide ISA 50 V2; LMQ: combination of neutral liposome, monophosphoryl lipid A, and *Quillaja saponaria* fraction 21; MPLA: *Bordetella pertussis* monophosphoryl lipid A; DDA: dimethyldioctadecylammonium bromide; AS04: TLR4 agonist monophosphoryl lipid A (MPLA) plus aluminum salt. ^c^ Via: Immunization route. IM: intramuscular; SC: subcutaneous; IP: intraperitoneal. ^d^ LD50: lethal dose equivalent to 50%. ^e^ Challenge type: HO: homologous serovar; HE: heterologous serovar. * Prime boost strategy: first dose with DNA vaccine and boost with subunit vaccine. ^f^ Percentual protection, the number of survivors/total is shown in parentheses; ^g^ Percentual of animals that achieved end-point criteria in the negative control group after challenge; ^h^ number of independent experiments performed; NA: Not Available.

## Data Availability

No new data were created or analyzed in this study. Data sharing is not applicable to this article.
